# Anterior cruciate ligament femoral side retained stump technique reduces enlargement of the femoral bone tunnel after anterior cruciate ligament reconstruction

**DOI:** 10.1186/s12891-024-07464-4

**Published:** 2024-05-14

**Authors:** Xiaobo Li, Hanlin Li, Jixian Su, Ran Ding

**Affiliations:** 1 Department of Orthopedics, General Hospital of Central Theater Command, 627 Wuluo Road, Wuchang District, Wuhan, Hubei Province China; 2 The First School of Clinical Medicine, Southern Medical University, Guangzhou, Guangdong Province China; 3 Clinical Medicine, Wuhan University of Science and Technology, 2 West Huangjiahu Road, Hongshan District, Wuhan, Hubei Province China

**Keywords:** Anterior cruciate ligament(ACL), Reconstruction, Remnant preservation, Femoral tunnel enlargement, Femoral side preservation technique

## Abstract

**Background:**

Enlargement of the bone tunnel has become an unavoidable early complication after anterior cruciate ligament (ACL) reconstruction, whether it is a single or double-bundle ACL reconstruction. Preservation of the ACL stump in ACL reconstruction reduces enlargement of the bone tunnel. The purpose of this study was to investigate the question of whether single-bundle ACL reconstruction using the ACL femoral side retained stump technique reduces enlargement of the femoral tunnel.

**Methods:**

Forty patients who underwent single-bundle reconstruction of the ACL were included in this study. The patients were categorized into a Remnant preservation group (Group R) and the Non-remnant preservation group (Group N). In the Remnant preservation group, a high-flexion femoral side retained stump technique was used intraoperatively for the establishment of the femoral side bone tunnel, and in the Non-remnant preservation group, the conventional femoral positioning method was used (we used a femoral positioning drill for localization and drilling of the femoral bone tunnel), and MRI of the operated knee joints was performed at 6 months postoperatively. We measured the internal diameter of the femoral bone tunnel at 5 mm from the intra-articular outlet of the femoral bone tunnel on an MRI scan image perpendicular to the femoral bone tunnel. The size of the tunnel was compared between the intraoperative drilling of the bone tunnel and the size of the bone tunnel at 6 months postoperatively. Postoperative clinical assessment was Lysholm score.

**Results:**

After a 6-month follow-up of 40 patients, the diameter of the femoral tunnel at a distance of 5 mm from the inner opening of the femoral tunnel was 10.96 ± 0.67 mm and 10.11 ± 0.62 mm in patients of group N and group R, respectively, and the difference was statistically significant (*P* < 0.05).The diameter of the femoral tunnel at 6 months postoperatively in group N and group R compared to the intraoperative bone tunnel increased by 2.58 ± 0.24 mm and 1.94 ± 0.31 mm, and the difference was statistically significant (*P* < 0.05).The femoral tunnel enlargement rates of group N and group R were 30.94 ± 3.00% and 24.02 ± 5.10%, respectively, and the differences were significant (*P* < 0.05).

**Conclusion:**

ACL femoral side retained stump technique does not sacrifice the ideal location of the femoral tunnel and is able to preserve the possible benefits of the ACL stump: reduced femoral tunnel enlargement.

## Background

The anterior cruciate ligament (ACL) is a crucial stabilising structure of the knee joint. Its primary function is to restrict the forward movement and internal rotation of the tibia. ACL injury results in anterolateral and rotational instability of the knee joint [1, 2]. The most frequent injury pattern is a non-contact mechanism during rotation and jump landings with the knee slightly flexed and valgus [3]. ACL injuries are a common type of knee injury, and ACL reconstruction is a frequently performed surgical procedure to effectively treat these injuries. The surgical outcome can be affected by both the position and enlargement of the bone tunnel [4]. Therefore, it is necessary to find ways to reduce bone tunnel enlargement after ACL surgery. In single-bundle ACL reconstruction, whether using autologous hamstring tendon, autologous patellar tendon, or allogeneic tendon, there is always some degree of postoperative enlargement of the bone tunnel [5–8]. The cause of postoperative enlargement of the bone tunnel is currently unknown. It is believed that the enlargement is caused by a combination of mechanical and biological factors [9, 10]. Mechanical factors that contribute to micromotion of the graft in the bone tunnel during ACL reconstruction with extracortical fixation in the femoral cortex include the ‘bungee effect’ or ‘windshield wiper effect’, localization of the bone tunnel, graft fixation, and active rehabilitation in the early postoperative period [10], localization of the bone tunnel [6], fixation of the graft [11–13], and active rehabilitation in the early postoperative period [14]. Biological factors include cytokines (interleukin 1, interleukin 6, tumor necrosis factor, nitric oxide, etc.) [15–17], inflammatory mediators, the type of graft, the quality of the bone, and osteoclastic necrosis resulting from the process of drilling bone tunnels [18–21].

In ACL reconstruction, preservation of the ACL stump has been shown to have many benefits: better ligamentization of the graft, better synovial coverage, blood supply to the graft, better proprioception, and better stability of the knee [22–24]. Additionally, preserving the ACL femoral stump has the added benefits of closing the femoral tunnel, preventing articular fluid leakage, facilitating tendon healing, and preventing tunnel enlargement [25]. Previous studies have shown that in double-bundle ACL reconstruction, the ACL stump can prevent the enlargement of the tibial bone tunnel. However, no scholars have studied the preservation of the femoral side stump in single-bundle ACL reconstruction to prevent the enlargement of the femoral bone tunnel.

The aim of this study was to investigate the correlation between femoral bone tunnel enlargement and ACL stump preservation after single-bundle ACL reconstruction using hamstring tendon. This is the first study to examine this relationship.

## Materials and methods

We collected 40 patients who underwent single-bundle ACL reconstruction from July 2019 to July 2022 at our hospital (Table 1). Inclusion criteria: initial ACL reconstruction, preoperative MRI and intraoperative arthroscopic confirmation of ACL rupture, positive Lachman test and/or axial shift test, clear history of trauma; ACL rupture within 6 months, use of autologous hamstring tendon for grafts, presence of ACL femoral side stump, informed consent of the patient, unilateral knee injury. Exclusion criteria: revision of ACL, multiple ligament injuries, combined intercondylar crest fracture, combined severe cartilage injury, combined other fractures. All patients signed an informed consent form, and the study was reviewed by the hospital ethics committee. All patients completed three-dimensional CT of the knee joint 3 days after surgery and MRI review of the knee joint 3 months after surgery. The femoral tunnel position is assessed using the lateral femoral condyle quadrant method, as described by Bernard et al [26].

**Table 1:** Demographic data for both groups

	Group N(*n* = 20)	Group R (*n* = 20)	*P* value
Age, year (mean ± SD)	26.7 ± 6.2	26.1 ± 5.2	n.s.
Gender (male/female)	16/4	18/2	n.s.
Involved side (right/left)	9/11	8/12	n.s.
Hight (cm)	175.1 ± 6.1	172.1 ± 5.1	n.s.
Weight (kg)	73.2 ± 7.8	73.5 ± 6.9	n.s.
BMI (kg/m^2^)	23.8 ± 1.4	24.8 ± 2.1	n.s.

**Fig. 1:**
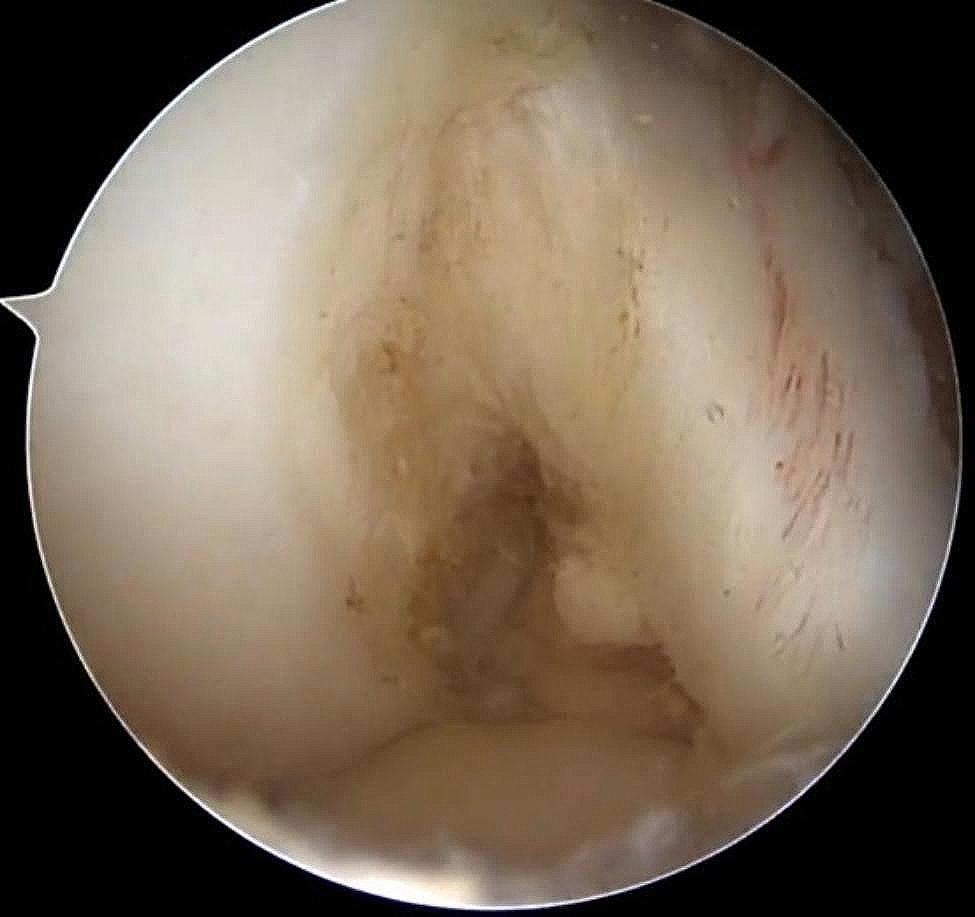
Arthroscopic visualization of the preserved ACL femoral lateral stump

Arthroscopy was performed through anteromedial (AM) and anterolateral (AL) manipulation and observation approaches. The integrity of the ACL was examined arthroscopically (Fig. 1), meniscal and knee cartilage injuries were examined, and intercondylar fossa stenosis was examined.

The choice of surgical protocol depended on the intraoperative record of the ACL remnants length measured by the primary surgeon and the attending physician using a tunnelling device (ACUFEX, Smith and Nephew Inc.). The ACL femoral stumps preservation technique was performed only when the primary surgeon and the attending physician agreed that the length of the ACL remnant was more than 25% of the original length of the intact ACL and that the ACL remnant was not partially disrupted at the tibial attachment site. These were included in Group R. This determination is consistent with earlier studies evaluating tibial tunnel [27]. If the length of the ACL remnant is less than 25% compared to the original length of the intact ACL, or if the tibial attachment is disrupted, the remnant will be debrided using an arthroscopic shaver or radiofrequency probe to well visualize the location of the femoral tunnel. These were included in Group N. Group R indicated preservation of the ACL stump and group N indicated no preservation of the ACL stump.

### Preparation and acquisition of grafts

Standard AM and AL incisions were taken. The patient was placed in the supine position and epidural or general anesthesia was administered. At 90° of flexion, the synovial membrane and part of the infrapatellar fat pad were removed, and the medial anterior end of the lateral femoral condyle was fully exposed to facilitate observation and positioning. The lateral femoral stump of the ACL has grouped arthroscopically (whether it belonged to group R or N) and then exited the arthroscope. An oblique skin incision was made on the medial tibial deviation to reveal the goosefoot tendon. The semitendinosus and gracilis tendons were then removed with a tendon extractor.

### Drilling of the femoral bone tunnel

After examining the lateral femoral footprint area using arthroscopy from the AL approach, the lateral ACL stump was preserved, and then the center of the footprint was marked with a plasma knife using the AM approach. The knee was flexed at 120°, and the plumb line between the lowest point of the cartilaginous margin of the lateral femoral condyle and the tibial plateau was used as a localization marker for the creation of the femoral bone tunnel (Fig. 2a and b). The appropriate depth of the bone tunnel was created based on the length of the bone tunnel and the length of the graft. During the preparation of the femoral bone tunnel, we preserved the stump of the femoral side of the ACL. After the femoral bone tunnel is established, we can see that the posterior wall of the bone tunnel is intact (Fig. 3). For group N, the femoral bone tunnel was drilled under the guidance of a femoral offset guide (ACUFEX, Smith and Nephew Inc.), which was placed through an AM portal. In the present study, the mean value of intraoperative femoral bone tunnel was 8.2 mm in group R and 8.3 mm in group N.

**Fig. 2a:**
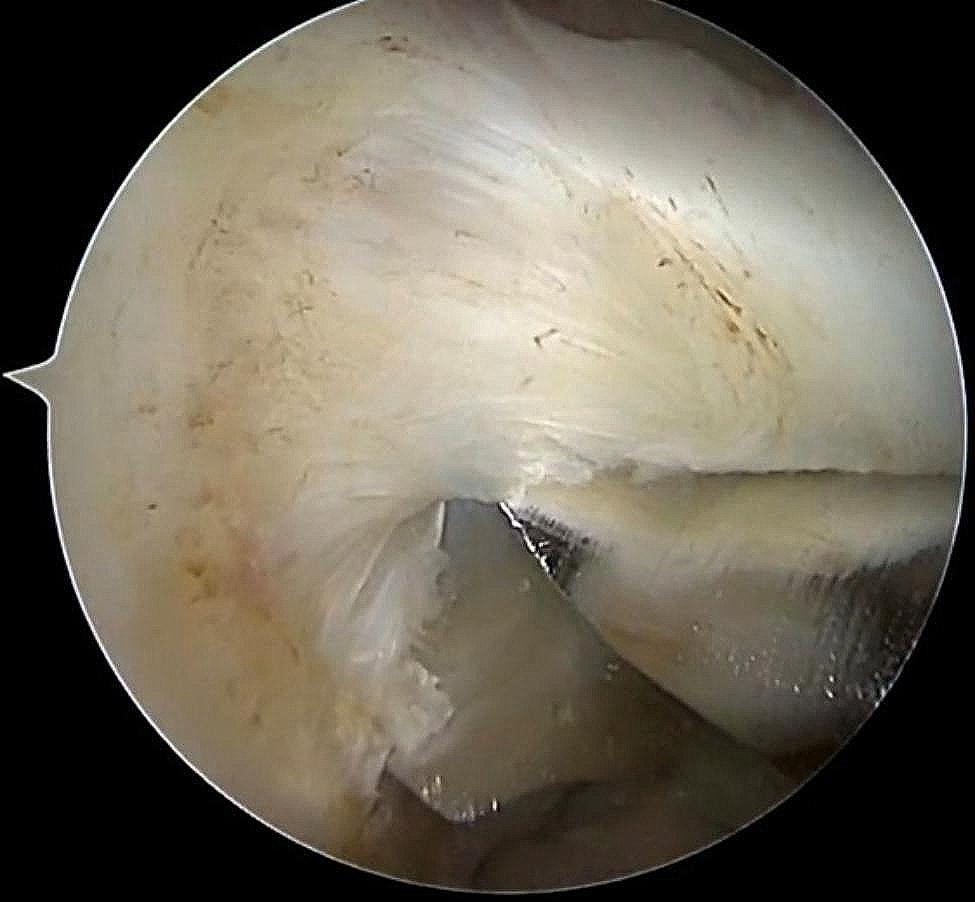
Establishment of the femoral bone tunnel for arthroscopic ACL reconstruction: a coarse hollow drill is shown, in which the anterior edge of the coarse hollow drill is tangent to the bifurcate ridge (the plumb line between the lowest point of the cartilaginous rim of the lateral condyle of the femur and the tibial plateau at 120 degrees of flexion), and the lower edge is approximately 2 mm from the tibial plateau

**Fig. 2b:**
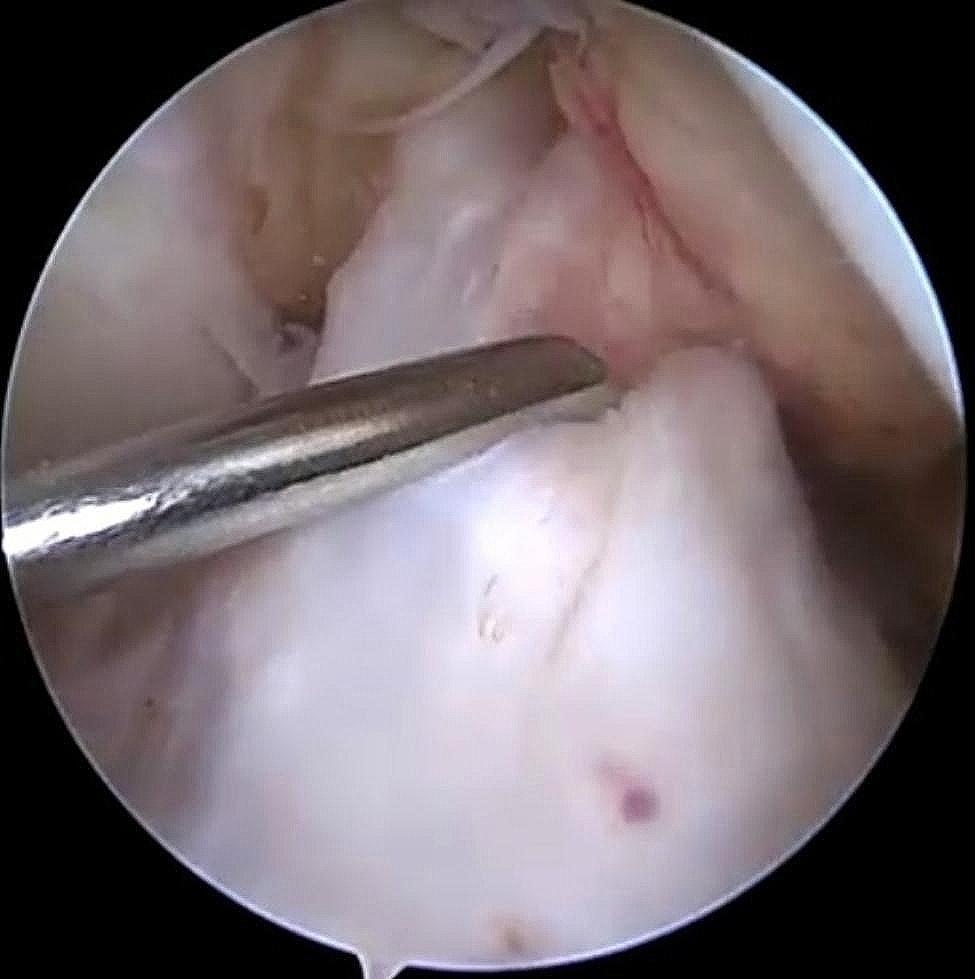
Arthroscopic exploration of the ACL: rupture of the ACL, but preservation of the ACL femoral side stump

**Fig. 3:**
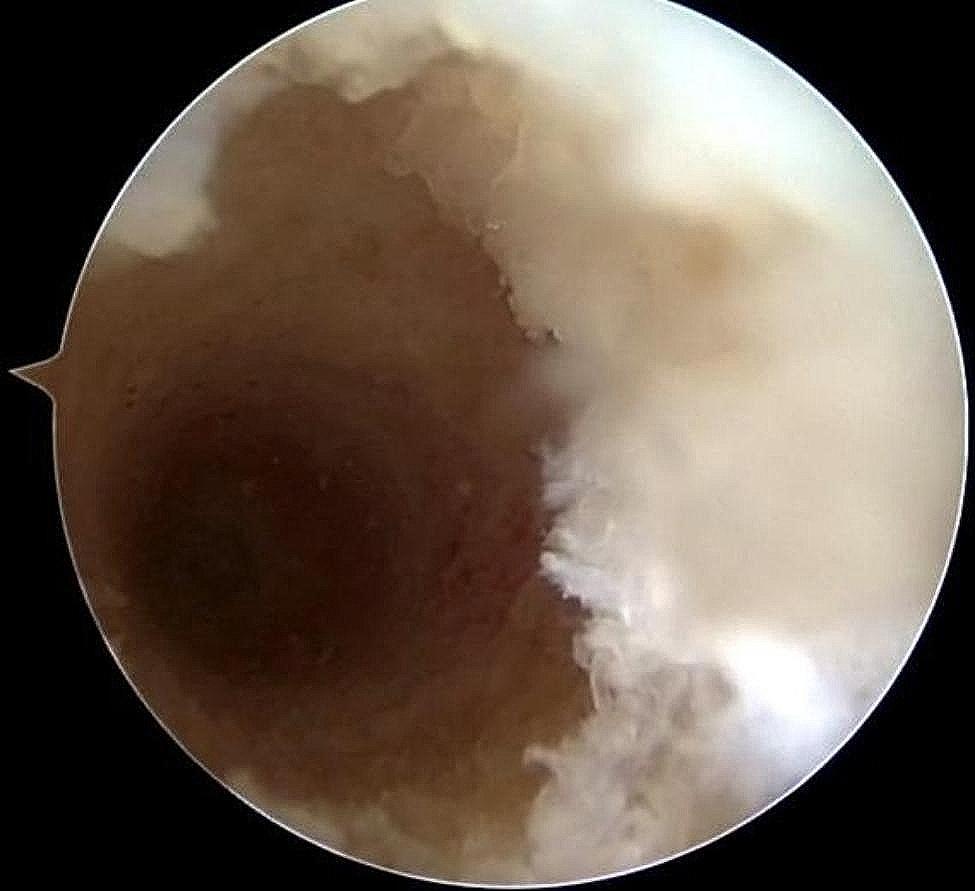
Femoral bone tunnel after arthroscopic ACL reconstruction: posterior wall of the tunnel is intact

### Drilling of the tibial tunnel and fixation of grafts

**Fig. 4:**
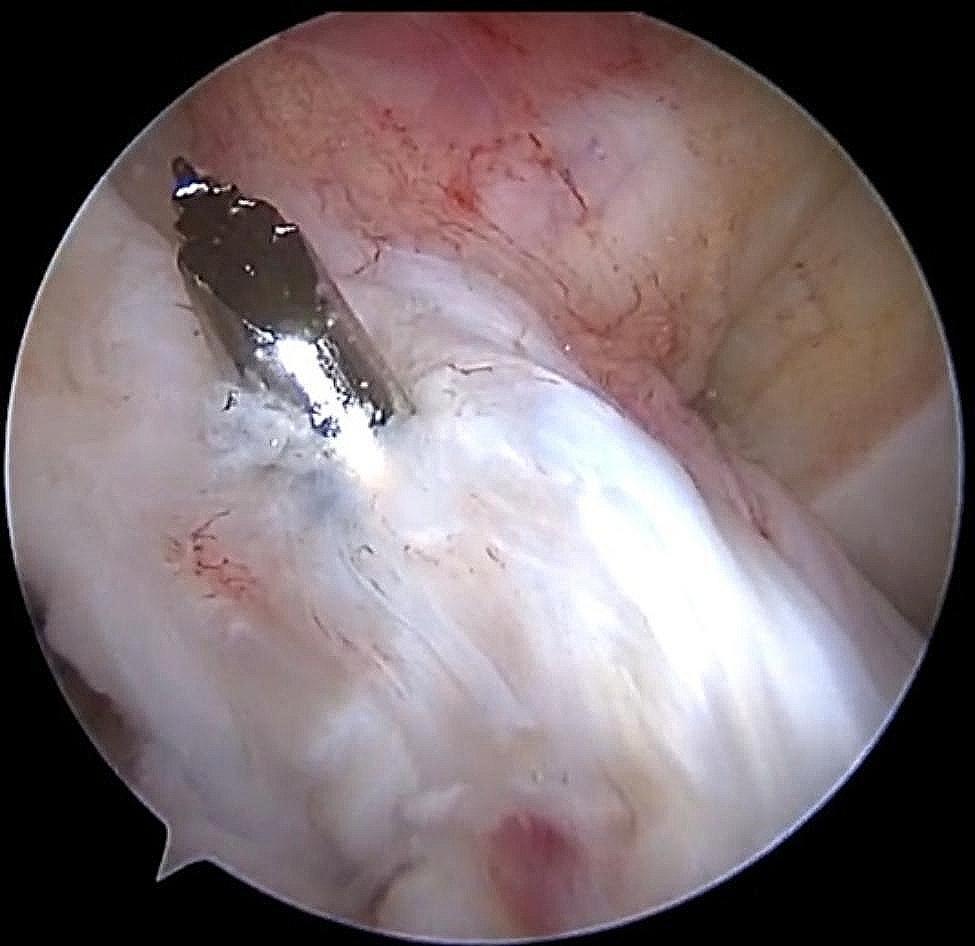
Arthroscopic ACL reconstruction: preservation of the tibial side remnant of the ACL

The tibial bone tunnel was created through the AM portal. In this study, we also preserved the tibial stump of the ACL (Fig. 4). Finally, the tendon was introduced into the bone tunnel from the tibial bone tunnel, confirming that the Endobutton was flipped over and well apposed at the outer portion of the osseous tunnel, and arthroscopy confirmed that the previous markings on the graft were flush with the entrance of the femoral osseous tunnel, indicating that the graft was fully entered into the femoral tunnel. An interference screw was placed with the knee flexed at 30° to maintain pressure on the graft [16]. Graft impingement was assessed during the procedure with the knee in full extension (Fig. 2).

### Postoperative rehabilitation

Early postoperative support immobilization in the knee extension position. At 2 weeks postoperatively, passive mobility of the joint reaches 90°, isometric muscle exercises are performed, and patellar mobility is practiced. 2–6 weeks after surgery, passive mobility reaches 120°, active mobility reaches 115°, lower limb muscle strength and proprioception exercises. 6–12 weeks after surgery, return to normal joint mobility, return to normal walking, lower limb proprioception, and flexibility exercises. 12–22 weeks after surgery, lower limb sensitivity training, lower limb strength returned to 80% of the healthy side, and special training gradually.

### Clinical evaluation

All postoperative patients were subjected to clinical examination and functional assessment 6 months after surgery. This mainly includes Lysholm score (Table 2).

**Table 2:** Comparison of Lysholm score

	Preoperative	Postoperative	*P* value
Group R	62.2 ± 5.7	92.05 ± 2.01	n.s
Group N	61.2 ± 6.4	92.30 ± 2.39	n.s
*P* value	n.s	n.s	-

### MRI evaluation

All patients’ knees were examined by MRI at 6 months postoperatively, and the enlargement of the bone tunnel was assessed by measuring the width of the bone tunnel on an MRI scan image perpendicular to the femoral bone tunnel (Fig. 5a). All bone tunnel measurements were performed by an experienced orthopedic surgeon. MRI data obtained at follow-up were compared with the diameter of the bone tunnel drilled intraoperatively. The values for the tunnel enlargement are reported in percentages using the following expression: (Enlargement/Intraoperative drill size) ×100% (Table 3).

**Table 3:** Measurements of femoral tunnel diameter

	Group *N*	Group *R*	*P* value
Intraoperative drill size	8.3 ± 0.5	8.2 ± 0.6	n.s.
Diameter at 5 mm from inlet (mm)	10.96 ± 0.67	10.11 ± 0.62	<0.05
Enlargement (mm)	2.58 ± 0.24	1.94 ± 0.31	<0.05
Enlargement rate (%)	30.94 ± 3.00	24.02 ± 5.10	<0.05

**Fig. 5a:**
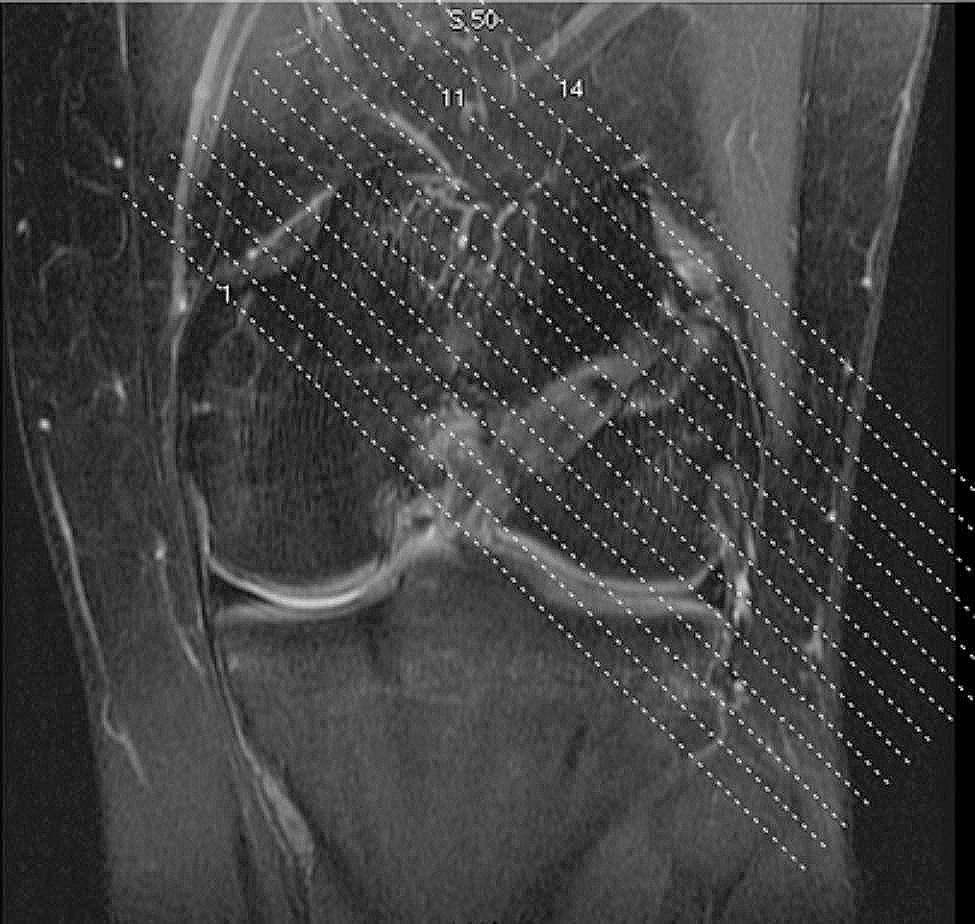
MRI of the knee 6 months after ACL reconstruction: This is a localized scan image perpendicular to the femoral bone tunnel of the knee joint(MRI has a scanning layer thickness of 5 mm)

**Fig. 5b:**
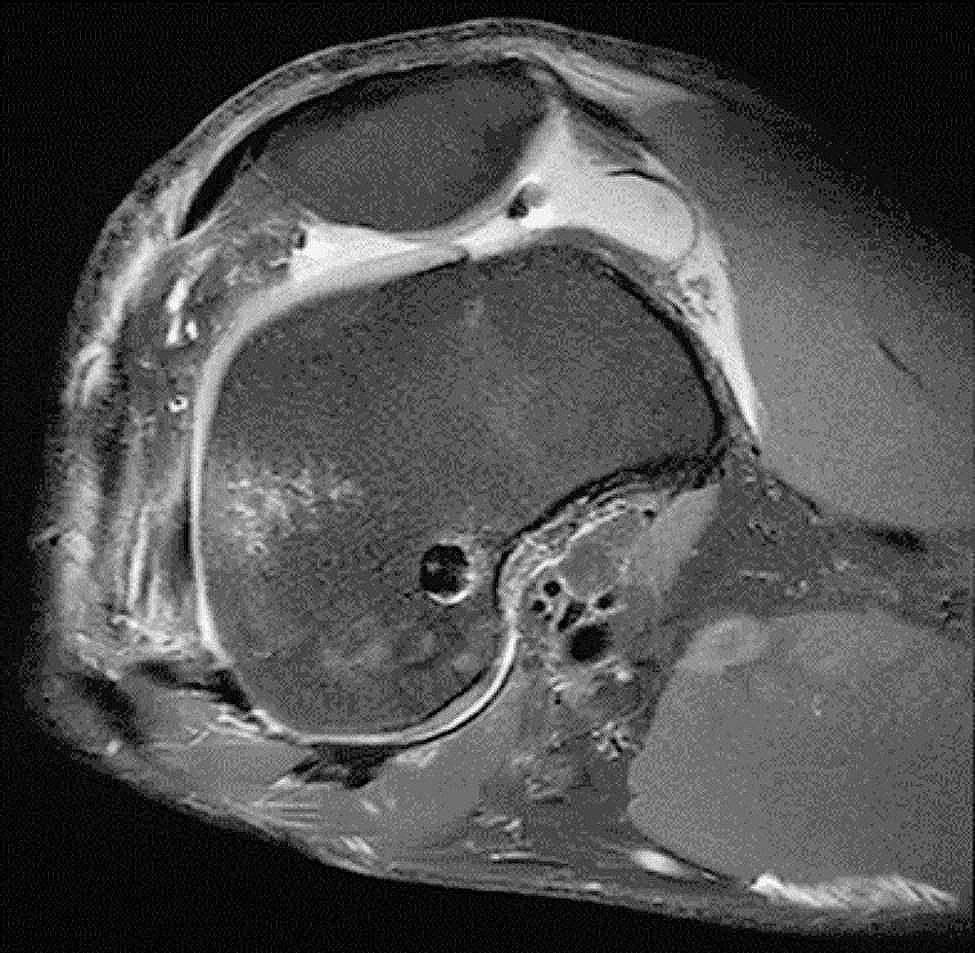
This is measurement of the internal diameter of the femoral tunnel at the level of an MRI scan perpendicular to the femoral tunnel(5 mm from the internal portion of the tunnel)

### Statistical analysis

Statistical analysis was performed using the SPSS software package version 25.0 (SPSS, Chicago, IL). Differences between means were calculated using analysis of variance (ANOVA) and paired samples t-test.

## Results

Femoral bone tunnel location.

**Fig. 6:**
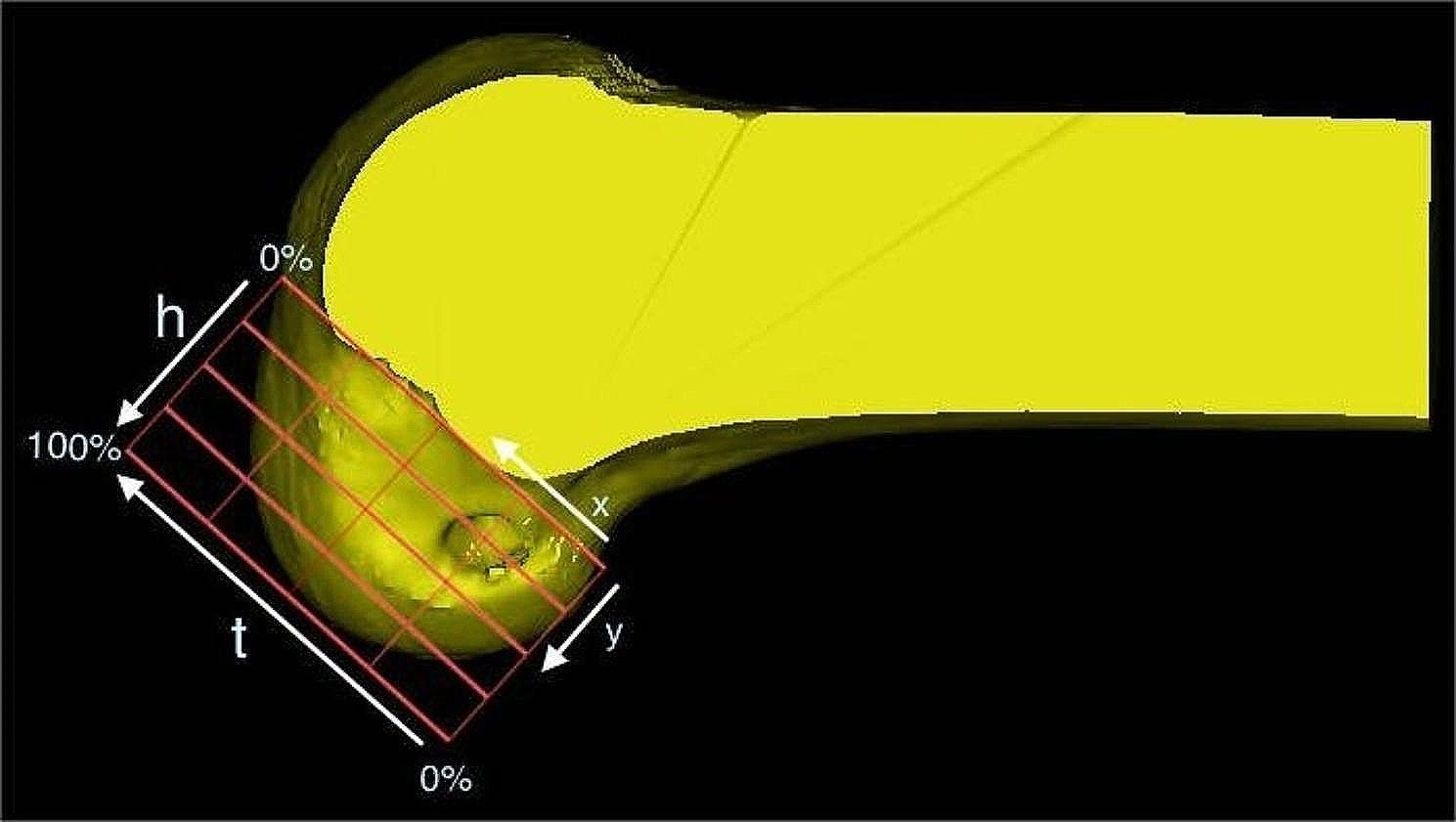
: **The femoral tunnel position can be evaluated using the quadrant method.***x*, distance from the center of the footprint to the proximal border along line *t*; *y*, distance from the center of the footprint to the Blumensaat line; *x* and *y* are expressed as percentages of *t* and *h*. *t*, total sagittal diameter of lateral condyle along the Blumensaat line; *h*, maximum intercondylar notch height

The bone tunnels were visualized using the 4 × 4 grid method proposed by Bernard et al [2](Fig. 6). The femoral bone tunnel locations were comparable between the two groups, with measurements of 24.3 ± 1.6 × 23.1 ± 2.6 (group R) and 25.8 ± 2.2 × 25.1 ± 4.0 (group N), as shown in Table 4. There was no significant difference in the location of the femoral bone tunnel between group R and group N.

**Table 4:** Tunnel locations in the femur

	Group *N*	Group *R*	*P* value
Femoral tunnel location(%)	25.8 ± 2.2 × 25.1 ± 4.0	24.5 ± 1.6 × 23.1 ± 2.6	
x	25.8 ± 2.2	24.5 ± 1.6	n.s.
y	25.1 ± 4.0	23.1 ± 2.6	n.s.

### Tunnel size during surgery and tunnel enlargement

The data of femoral bone tunnel measurements and enlargement rate in all patients are shown in Table 3. As can be seen in Table 2, there are 20 knees in group R and 20 knees in group N. The rate of femoral bone tunnel enlargement (*P* = 0.0 00) was significantly lower in group R compared to group N. The difference in Lysholm scores was not statistically significant in either group R or N.

## Discussion

The most important finding of this study is that ACL reconstruction using the ACL femoral side remnant-preservation technique resulted in less enlargement of the bone tunnel in the femur, thus making the late ACL revision surgery easier while preserving the benefits of the ACL stump.

ACL reconstruction is a standard procedure for the treatment of ACL injuries and is currently obtaining better clinical results, however, the related postoperative complications have not been resolved. Postoperative bone tunnel enlargement is a common problem in ACL reconstruction [28, 29], occurring mainly within 6 months after ACL reconstruction [29]. Although bone tunnel widening has been reported to have no significant correlation on clinical outcomes [30], bone tunnel widening can move the bone tunnel to a non-anatomic location [31] and affect the treatment strategy for ACL revision surgery [29, 32]. However, the specific etiology of tunnel widening remains unclear. The mechanism of the occurrence of bone tunnel enlargement after ACL reconstruction is still unclear. The occurrence of tunnel enlargement may be multifactorial, and most scholars believe that channel enlargement is caused by the complex interaction of mechanical and biological factors[57].The known mechanical factors include the following: first, longitudinal movement (“bungee effect”) and transverse movement (“windshield wiper effect”) due to micromotion of the graft in the bone tunnel [33, 34]. Several previous in vitro experimental studies have pointed out that the site of fixation of the graft affects the movement of the graft in the bone tunnel, i.e., the longer the distance between the points of fixation of the graft, the significantly greater the longitudinal and transverse movement of the graft [34]. This is due to the fact that patients begin joint range-of-motion exercises and weight-bearing walking before tendon-bone healing is complete [33, 35, 36]. Second, the positioning of the bone tunnel also affects the enlargement of the tunnel [37, 38]. Higher and more anterior femoral osseous tract positioning results in more femoral tunnel enlargement [38]. In the 40 patients in this study, all the femoral and tibial bone tunnels were in the proper position. In addition, intraoperative drill use, localized thermal necrosis of the bone, and nonspecific inflammatory responses may contribute to early tunnel enlargement after ACL reconstruction [51]. Third, the fixation mode of the graft [11, 12]. Currently, in ACL reconstruction, there are two main fixation methods for the femoral side, one is extrusion interface screw fixation; the other is cortical suspension titanium plate, which is divided into adjustable tabs and fixed tabs. Either type of fixation ultimately results in varying degrees of enlargement of the bone tunnel. The findings of some investigators have shown [16] that in single-bundle ACL reconstruction, there is a more pronounced widening of the femoral bone tunnel with fixation using fixation tabs compared to extruded interface screws because the closer the femoral end fixation position is to the joint line, which leads to less widening of the femoral tunnel. So the closer the fixation is to the joint, the lower the incidence of bone tunnel widening. In the present study, all patients with lateral femoral grafts underwent cortical external fixation with Endobutton-suspended titanium plates, assuming that the mechanical effects of bungee and windshield wipers resulted in more widening of the tunnel at the exit. Fourth, an aggressive rehabilitation program [50]. Fifth, the diameter of the graft. The Tendon graft diameter was kept at 8 ∼ 9 mm [39]. In this study, all patients underwent single-bundle ACL reconstruction, and too large a diameter of the graft can cause excessive stress loading on the edges of the bone tunnel thereby causing abnormal enlargement of the bone tunnel [40].

Most studies have shown that enlargement of the bone tunnel after ACL reconstruction has no effect on clinical outcomes, but enlarged bone tunnels may pose additional difficulties in ACL revision surgery. Recent studies have shown that tunnel enlargement is accompanied by changes in the anatomical position of the tunnel [31, 41], which may be related to unevenly distributed mechanical stresses on the tunnel wall [42]. Tunnel enlargement results in an eccentric shift of the femoral tunnel position in the anterior and distal directions. As a result, the mechanical traction of the ACL graft constantly generates eccentric stresses on the tunnel wall, leading to tunnel widening [43]. Moreover, it has been shown that there is an increase in case changes in the meniscus after ACL revision surgery [44], which may lead to more severe osteoarthritis. It has been estimated that up to 60% of patients undergoing ACL revision reconstruction will develop osteoarthritis of the knee within approximately 6 years [45]. This risk is twice that of initial ACL reconstruction surgery [46]. ACL revision surgery is not simply an ACL reconstruction procedure, we need to be aware of the causes of ACL graft failure. For ACL revision surgery, we also need a detailed preoperative plan, to fully understand the details of the initial ACL reconstruction surgery, such as the type of graft, the diameter of the bone channel, and the fixation method. Emphasis should be placed on analyzing the reasons for the failure of the initial ACL surgery. The choice of revision graft for ACL reconstruction plays an important role in the success of the surgery. Because of the high re-tear rate and poor clinical outcome of homografts [47, 48], the use of homografts should be avoided as much as possible. The selected graft should be of sufficient diameter. In revision surgery, graft fixation is as important as graft selection. Graft fixation should be firm and reliable, and double fixation is necessary when the bone quality or quantity of bone in the bone tunnel is poor [49]. For bone tunnel enlargement, revision of the ACL may require either a one-stage revision or a two-stage revision. Most ACL reconstructions can be accomplished with a one-stage procedure. The ideal indication for a one-stage revision would be complete nonanatomic positioning of the bone tunnel and minimal enlargement of the old bone tunnel. In this case, a new tunnel can be drilled at the anatomic footprint of the ACL while maintaining an adequate bone bridge between the old and new bone tunnels. Next is the fully anatomically positioned old tunnel with no or minimal enlargement, which can be achieved by enlarging the old tunnel and selecting a larger graft. If the bone tunnel is positioned close to the anatomical structure and the enlargement of the bone channel is between 14 and 16 mm, it can be managed in one stage [50]. Another approach to address femoral bone tunnel enlargement is the use of over-the-top position reconstruction techniques [51]. The management of tunnel enlargement is a more problematic issue if the tunnel diameter is > 14–16 mm or if the tunnel is positioned to ride across an anatomic stop [49]. In the first stage of the revision procedure, the interference screws are removed and the enlarged femoral bone tunnel is grafted. At the completion of the first stage, the sclerotic bone tunnel wall will be fresh with a drill to promote healing of the implant. The second stage revision surgery is usually performed after 4–6 months [49]. On the one hand, this may leave the patient’s knee in an unstable state for a prolonged period of time, which can increase secondary injuries such as meniscus. On the other hand, the lack of movement of the knee joint while waiting for the second stage of surgery can lead to knee stiffness and atrophy of the muscles around the knee. Secondly, undergoing more than one surgery can result in an unpleasant experience for the patient and an increase in medical expenses. Therefore, widening of the bone tunnel may further complicate revision surgery in these knees. Therefore, surgeons should fully recognize the adverse effects of bone tunnel widening on ACL revision surgery and take measures to reduce bone tunnel widening after ACL reconstruction. In this study, we adopted the ACL femoral lateral disability preservation technique to prevent femoral bone tunnel widening, thus avoiding the unfavorable factors associated with bone tunnel widening.

Preservation of the ACL stump has both advantages and disadvantages. In ACL reconstruction, the prevailing view is to preserve the tibial ACL stump. As for the femoral side stump, most of the operators chose to shave off the ACL femoral side remnant because of the need to visualization of the bony landmarks of the lateral femoral condyle for accurate localisation. In the present study, we preserved the ACL femoral side stump. The advantages of preserving the stump have been widely studied. Currently, preservation of the ACL stump is an important method to prevent enlargement of the bone tunnel.

Although the focus of this study was on the femoral side to preserve the femoral side ACL stump, we also preserved the tibial side ACL stump. In addition, preservation of the stump has been shown to be a risk factor for the development of arthrofibrosis, cyclops lesion and intercondylar fossa impingement of the knee [52].

Most of the current studies have not yet agreed on the time of occurrence of bone tunnel enlargement after ACL reconstruction. Some scholars believe that postoperative bone tunnel enlargement after ACL reconstruction occurs mainly at 3 months postoperatively [51], while others believe that bone tunnel enlargement occurs within 3 months postoperatively and can last up to 9 months [53]. It has also been shown that the period of maximum bone tunnel enlargement is 10 months postoperatively and there is no further significant increase in the following period [54]. However, tunnel enlargement is usually maximized at 24 weeks postoperatively, as reported in a related study [55]. In summary, we finally chose the patient’s MRI at 24 weeks postoperatively for bone tunnel measurements to assess postoperative tunnel enlargement.

It has been shown that enlargement of the bone tunnel mainly occurs within 5 mm from the exit of the bone tunnel [54] and that the error in measuring the tunnel diameter on the medial side of the femoral condyles due to the inclination of the entrance was eliminated by measuring the tunnel diameter at 5 mm from the entrance. Therefore, in this study, we set the measurement of the bone tunnel at 5 mm from the inner opening of the femoral tunnel as a uniform standard for comparison purposes.

Tibial tunnel widening was not assessed in this study. All patients were internally fixed on the tibial side with bioabsorbable interference screws.

Our current study found that preservation of the ACL femoral remnant in ACL reconstruction was associated with a reduced incidence of femoral tunnel enlargement, but it did not seem to affect the clinical outcomes on the Lysholm score. The same results were reported by Hong et al [56]. They performed ACL reconstruction using a technique that preserved the remnant and showed that this technique did not improve postoperative knee function scores or laxity.

This study also has some limitations. First, the number of patients in our study was small. Follow-up studies would like to include more patients for further study. Second, immediate postoperative MRI was missing for comparison. Third, the follow-up time of the current study was only 6 months. Follow-up studies include MRIs at 1 year, and 2 years postoperatively. Fourth, in the current study, grafts we used only autologous hamstring tendons and lacked comparison with the use of bone-tendon-bone grafts. This is because previous studies have shown that tunnel enlargement after ACL reconstruction using hamstring tendon grafts is greater than using patellar tendon grafts. Finally, we did not assess the bone density of the patients, as this may also affect bone tunnel enlargement.

The present study is clinically relevant because it demonstrates the changes in the femoral bone tunnel after ACL reconstruction with or without preservation of the ACL femoral remnant, and it also illustrates the advantages of the ACL femoral side retained stump technique in ACL reconstruction. The ACL femoral side retained stump technique can reduce the enlargement of the femoral bone tunnel and can reduce some unnecessary troubles for future ACL revision surgery. At present, the remnant-preserving technique has been favoured by surgeons at home and abroad.

## Conclusion

This study demonstrated no significant difference in femoral tunnel localization between the Group N and Group R. It is therefore concluded that the ACL femoral side retained stump technique does not sacrifice the ideal location of the femoral tunnel and is able to preserve the possible benefits of the ACL stump: reduced femoral tunnel enlargement.

## Data Availability

The datasets used or analyzed during the current study are available from the corresponding author upon reasonable request.

## Declarations

### Founding

This work was supported by the 2020 Natural Science Foundation of Hubei Province (2020CFB570).

## Abbreviations

ACL: Anterior cruciate ligament
MRI: Magnetic resonance imaging
BMI: Body mass index

## References

[1] Zantop T, Herbort M, Raschke MJ, Fu FH, Petersen W (2007). The role of the anteromedial and posterolateral bundles of the anterior cruciate ligament in anterior tibial translation and internal rotation. Am J Sports Med.

[2] Musahl V, Kopf S, Rabuck S, Becker R, van der Merwe W, Zaffagnini S, Fu FH, Karlsson J (2012). Rotatory knee laxity tests and the pivot shift as tools for ACL treatment algorithm. Knee Surg Sports Traumatol Arthrosc.

[3] Alentorn-Geli E, Myer GD, Silvers HJ, Samitier G, Romero D, Lázaro-Haro C, Cugat R (2009). Prevention of non-contact anterior cruciate ligament injuries in soccer players. Part 1: mechanisms of injury and underlying risk factors. Knee Surg Sports Traumatol Arthrosc.

[4] Salmon L, Russell V, Musgrove T, Pinczewski L, Refshauge K (2005). Incidence and risk factors for graft rupture and contralateral rupture after anterior cruciate ligament reconstruction. Arthroscopy.

[5] Buelow JU, Siebold R, Ellermann A (2002). A prospective evaluation of tunnel enlargement in anterior cruciate ligament reconstruction with hamstrings: extracortical versus anatomical fixation. Knee Surg Sports Traumatol Arthrosc.

[6] Clatworthy MG, Annear P, Bulow JU, Bartlett RJ (1999). Tunnel widening in anterior cruciate ligament reconstruction: a prospective evaluation of hamstring and patella tendon grafts. Knee Surg Sports Traumatol Arthrosc.

[7] L’Insalata JC, Klatt B, Fu FH, Harner CD (1997). Tunnel expansion following anterior cruciate ligament reconstruction: a comparison of hamstring and patellar tendon autografts. Knee Surg Sports Traumatol Arthrosc.

[8] Zijl JA, Kleipool AE, Willems WJ (2000). Comparison of tibial tunnel enlargement after anterior cruciate ligament reconstruction using patellar tendon autograft or allograft. Am J Sports Med.

[9] Yanagisawa S, Kimura M, Hagiwara K, Ogoshi A, Nakagawa T, Shiozawa H, Ohsawa T, Chikuda H (2018). The remnant preservation technique reduces the amount of bone tunnel enlargement following anterior cruciate ligament reconstruction. Knee Surg Sports Traumatol Arthrosc.

[10] Nebelung W, Becker R, Merkel M, Röpke M (1998). Bone tunnel enlargement after anterior cruciate ligament reconstruction with semitendinosus tendon using Endobutton fixation on the femoral side. Arthroscopy.

[11] Jagodzinski M, Foerstemann T, Mall G, Krettek C, Bosch U, Paessler HH (2005). Analysis of forces of ACL reconstructions at the tunnel entrance: is tunnel enlargement a biomechanical problem?. J Biomech.

[12] Fauno P, Kaalund S (2005). Tunnel widening after hamstring anterior cruciate ligament reconstruction is influenced by the type of graft fixation used: a prospective randomized study. Arthroscopy.

[13] Giron F, Aglietti P, Cuomo P, Mondanelli N, Ciardullo A (2005). Anterior cruciate ligament reconstruction with double-looped Semitendinosus and Gracilis tendon graft directly fixed to cortical bone: 5-year results. Knee Surg Sports Traumatol Arthrosc.

[14] Vadalà A, Iorio R, De Carli A, Argento G, Di Sanzo V, Conteduca F, Ferretti A (2007). The effect of accelerated, brace free, rehabilitation on bone tunnel enlargement after ACL reconstruction using hamstring tendons: a CT study. Knee Surg Sports Traumatol Arthrosc.

[15] Caborn DN, Johnson BM (1993). The natural history of the anterior cruciate ligament-deficient knee. A review. Clin Sports Med.

[16] Zysk SP, Fraunberger P, Veihelmann A, Dörger M, Kalteis T, Maier M, Pellengahr C, Refior HJ (2004). Tunnel enlargement and changes in synovial fluid cytokine profile following anterior cruciate ligament reconstruction with patellar tendon and hamstring tendon autografts. Knee Surg Sports Traumatol Arthrosc.

[17] Cohen J (1998). The role of access of joint fluid to bone in periarticular osteolysis. A report of four cases. J Bone Joint Surg Am.

[18] Aga C, Wilson KJ, Johansen S, Dornan G, La Prade RF, Engebretsen L (2017). Tunnel widening in single- versus double-bundle anterior cruciate ligament reconstructed knees. Knee Surg Sports Traumatol Arthrosc.

[19] Vergis A, Gillquist J (1995). Graft failure in intra-articular anterior cruciate ligament reconstructions: a review of the literature. Arthroscopy.

[20] Silva A, Sampaio R, Pinto E (2010). Femoral tunnel enlargement after anatomic ACL reconstruction: a biological problem?. Knee Surg Sports Traumatol Arthrosc.

[21] Kobayashi M, Nakagawa Y, Suzuki T, Okudaira S, Nakamura T (2006). A retrospective review of bone tunnel enlargement after anterior cruciate ligament reconstruction with hamstring tendons fixed with a metal round cannulated interference screw in the femur. Arthroscopy.

[22] Takahashi T, Kimura M, Hagiwara K, Ohsawa T, Takeshita K (2019). The Effect of Remnant tissue preservation in anatomic double-bundle ACL Reconstruction on knee Stability and Graft Maturation. J Knee Surg.

[23] Bali K, Dhillon MS, Vasistha RK, Kakkar N, Chana R, Prabhakar S (2012). Efficacy of immunohistological methods in detecting functionally viable mechanoreceptors in the remnant stumps of injured anterior cruciate ligaments and its clinical importance. Knee Surg Sports Traumatol Arthrosc.

[24] Sonnery-Cottet B, Lavoie F, Ogassawara R, Scussiato RG, Kidder JF, Chambat P (2010). Selective anteromedial bundle reconstruction in partial ACL tears: a series of 36 patients with mean 24 months follow-up. Knee Surg Sports Traumatol Arthrosc.

[25] Masuda T, Kondo E, Onodera J, Kitamura N, Inoue M, Nakamura E, Yagi T, Iwasaki N, Yasuda K (2018). Effects of Remnant tissue preservation on tunnel enlargement after anatomic double-bundle Anterior Cruciate Ligament Reconstruction using the Hamstring Tendon. Orthop J Sports Med.

[26] Bernard M, Hertel P, Hornung H, Cierpinski T (1997). Femoral insertion of the ACL. Radiographic quadrant method. Am J Knee Surg.

[27] Kosy JD, Walmsley K, Gordon EA, Heddon SV, Anaspure R, Schranz PJ, Mandalia VI (2021). Remnant preservation does not affect accuracy of tibial tunnel positioning in single-bundle ACL reconstruction. Knee Surg Sports Traumatol Arthrosc.

[28] Bhullar R, Habib A, Zhang K, de Sa D, Horner NS, Duong A, Simunovic N, Espregueira-Mendes J, Ayeni OR (2019). Tunnel osteolysis post-ACL reconstruction: a systematic review examining select diagnostic modalities, treatment options and rehabilitation protocols. Knee Surg Sports Traumatol Arthrosc.

[29] Chiang ER, Chen KH, Chih-Chang LA, Wang ST, Wu HT, Ma HL, Chang MC, Liu CL, Chen TH (2019). Comparison of tunnel enlargement and clinical outcome between Bioabsorbable Interference screws and cortical button-post fixation in arthroscopic double-bundle Anterior Cruciate Ligament Reconstruction: a prospective, randomized study with a Minimum Follow-Up of 2 years. Arthroscopy.

[30] Taketomi S, Inui H, Yamagami R, Kawaguchi K, Nakazato K, Kono K, Kawata M, Nakagawa T, Tanaka S (2020). Length of the Tendon within the tibial tunnel affects tibial tunnel widening following anatomic Anterior Cruciate Ligament Reconstruction using a bone-patellar tendon-bone graft. J Knee Surg.

[31] Lee DK, Kim JH, Lee SS, Lee BH, Kim H, Kim J, Wang JH (2021). Femoral tunnel widening after double-bundle Anterior Cruciate Ligament Reconstruction with Hamstring Autograft produces a small shift of the tunnel position in the anterior and distal direction: computed tomography-based Retrospective Cohort Analysis. Arthroscopy.

[32] Wolfson TS, Mannino B, Owens BD, Waterman BR, Alaia MJ (2023). Tunnel management in Revision Anterior Cruciate Ligament Reconstruction: current concepts. Am J Sports Med.

[33] Höher J, Möller HD, Fu FH (1998). Bone tunnel enlargement after anterior cruciate ligament reconstruction: fact or fiction?. Knee Surg Sports Traumatol Arthrosc.

[34] Tsuda E, Fukuda Y, Loh JC, Debski RE, Fu FH, Woo SL (2002). The effect of soft-tissue graft fixation in anterior cruciate ligament reconstruction on graft-tunnel motion under anterior tibial loading. Arthroscopy.

[35] Kim SJ, Bae JH, Song SH, Lim HC (2013). Bone tunnel widening with autogenous bone plugs versus bioabsorbable interference screws for secondary fixation in ACL reconstruction. J Bone Joint Surg Am.

[36] Wilson TC, Kantaras A, Atay A, Johnson DL (2004). Tunnel enlargement after anterior cruciate ligament surgery. Am J Sports Med.

[37] Segawa H, Omori G, Tomita S, Koga Y (2001). Bone tunnel enlargement after anterior cruciate ligament reconstruction using hamstring tendons. Knee Surg Sports Traumatol Arthrosc.

[38] Ko YW, Rhee SJ, Kim IW, Yoo JD (2015). The correlation of tunnel position, orientation and tunnel enlargement in outside-in single-bundle Anterior Cruciate Ligament Reconstruction. Knee Surg Relat Res.

[39] Lee DC, Shon OJ, Kwack BH, Lee SJ (2013). Proprioception and clinical results of anterolateral single-bundle posterior cruciate ligament reconstruction with remnant preservation. Knee Surg Relat Res.

[40] Kawaguchi Y, Kondo E, Kitamura N, Kai S, Inoue M, Yasuda K (2011). Comparisons of femoral tunnel enlargement in 169 patients between single-bundle and anatomic double-bundle anterior cruciate ligament reconstructions with hamstring tendon grafts. Knee Surg Sports Traumatol Arthrosc.

[41] Li H, Liu S, Sun Y, Li H, Chen S, Chen J (2019). Influence of Graft bending Angle on Graft Maturation, the femoral tunnel, and functional outcomes by 12 months after Anterior Cruciate Ligament Reconstruction. Orthop J Sports Med.

[42] Hoshino Y, Kuroda R, Nishizawa Y, Nakano N, Nagai K, Araki D, Oka S, Kawaguchi S, Nagamune K, Kurosaka M (2018). Stress distribution is deviated around the aperture of the femoral tunnel in the anatomic anterior cruciate ligament reconstruction. Knee Surg Sports Traumatol Arthrosc.

[43] Liu D, Cai ZJ, Lu WH, Pan LY, Yang YT, Li YS, Xiao WF (2023). Eccentrically widened bone tunnels after all-inside anterior cruciate ligament reconstruction: a computed tomography and three-dimensional model-based analysis. Knee Surg Sports Traumatol Arthrosc.

[44] Thomas NP, Kankate R, Wandless F, Pandit H (2005). Revision anterior cruciate ligament reconstruction using a 2-stage technique with bone grafting of the tibial tunnel. Am J Sports Med.

[45] Grassi A, Kim C, Marcheggiani MG, Zaffagnini S, Amendola A (2017). What is the mid-term failure rate of Revision ACL Reconstruction? A systematic review. Clin Orthop Relat Res.

[46] Grassi A, Ardern CL, Marcheggiani MG, Neri MP, Marcacci M, Zaffagnini S (2016). Does revision ACL reconstruction measure up to primary surgery? A meta-analysis comparing patient-reported and clinician-reported outcomes, and radiographic results. Br J Sports Med.

[47] Ding DY, Zhang AL, Allen CR, Anderson AF, Cooper DE, Deberardino TM, Dunn WR, Haas AK, Huston LJ, Lantz B (2017). Subsequent surgery after Revision Anterior Cruciate Ligament Reconstruction: Rates and Risk factors from a Multicenter Cohort. Am J Sports Med.

[48] Effect of graft choice on the outcome (2014). Of revision anterior cruciate ligament reconstruction in the Multicenter ACL Revision Study (MARS) Cohort. Am J Sports Med.

[49] Richter DL, Werner BC, Miller MD (2017). Surgical pearls in Revision Anterior Cruciate ligament surgery: when must I Stage?. Clin Sports Med.

[50] Maak TG, Voos JE, Wickiewicz TL, Warren RF (2010). Tunnel widening in revision anterior cruciate ligament reconstruction. J Am Acad Orthop Surg.

[51] Cheatham SA, Johnson DL (2013). Anticipating problems unique to revision ACL surgery. Sports Med Arthrosc Rev.

[52] Nayak M, Nag HL, Gaba S, Nag TC, Sharma S (2018). Quantitative correlation of mechanoreceptors in tibial remnant of ruptured human anterior cruciate ligament with duration of injury and its significance: an immunohistochemistry-based observational study. J Orthop Traumatol.

[53] Drogset JO, Grøntvedt T, Myhr G (2006). Magnetic resonance imaging analysis of bioabsorbable interference screws used for fixation of bone-patellar tendon-bone autografts in endoscopic reconstruction of the anterior cruciate ligament. Am J Sports Med.

[54] Tachibana Y, Mae T, Shino K, Kanamoto T, Sugamoto K, Yoshikawa H, Nakata K (2015). Morphological changes in femoral tunnels after anatomic anterior cruciate ligament reconstruction. Knee Surg Sports Traumatol Arthrosc.

[55] Weber AE, Delos D, Oltean HN, Vadasdi K, Cavanaugh J, Potter HG, Rodeo SA (2015). Tibial and femoral tunnel changes after ACL Reconstruction: a prospective 2-Year longitudinal MRI study. Am J Sports Med.

[56] Hong L, Li X, Wang XS, Zhang H, Feng H (2011). [Arthroscopic anterior cruciate ligament reconstruction with remnant preservation: a prospective comparison study]. Zhonghua Wai Ke Za Zhi.

